# Supplementary material for: Proline Dehydrogenase (PRODH) Is Expressed in Lung Adenocarcinoma and Modulates Cell Survival and 3D Growth by Inducing Cellular Senescence
Source: Int J Mol Sci. 2024 Jan 5;25(2):714. doi: 10.3390/ijms25020714 (PMC10815008; doi:10.3390/ijms25020714)
Supplement: Supplementary file 1 [file ijms-25-00714-s001.zip › Supplementary figures S1-S3 raw data western blots.pdf]

Figure S1.
Raw data of the immunoblots presented in Figure 2, left panel

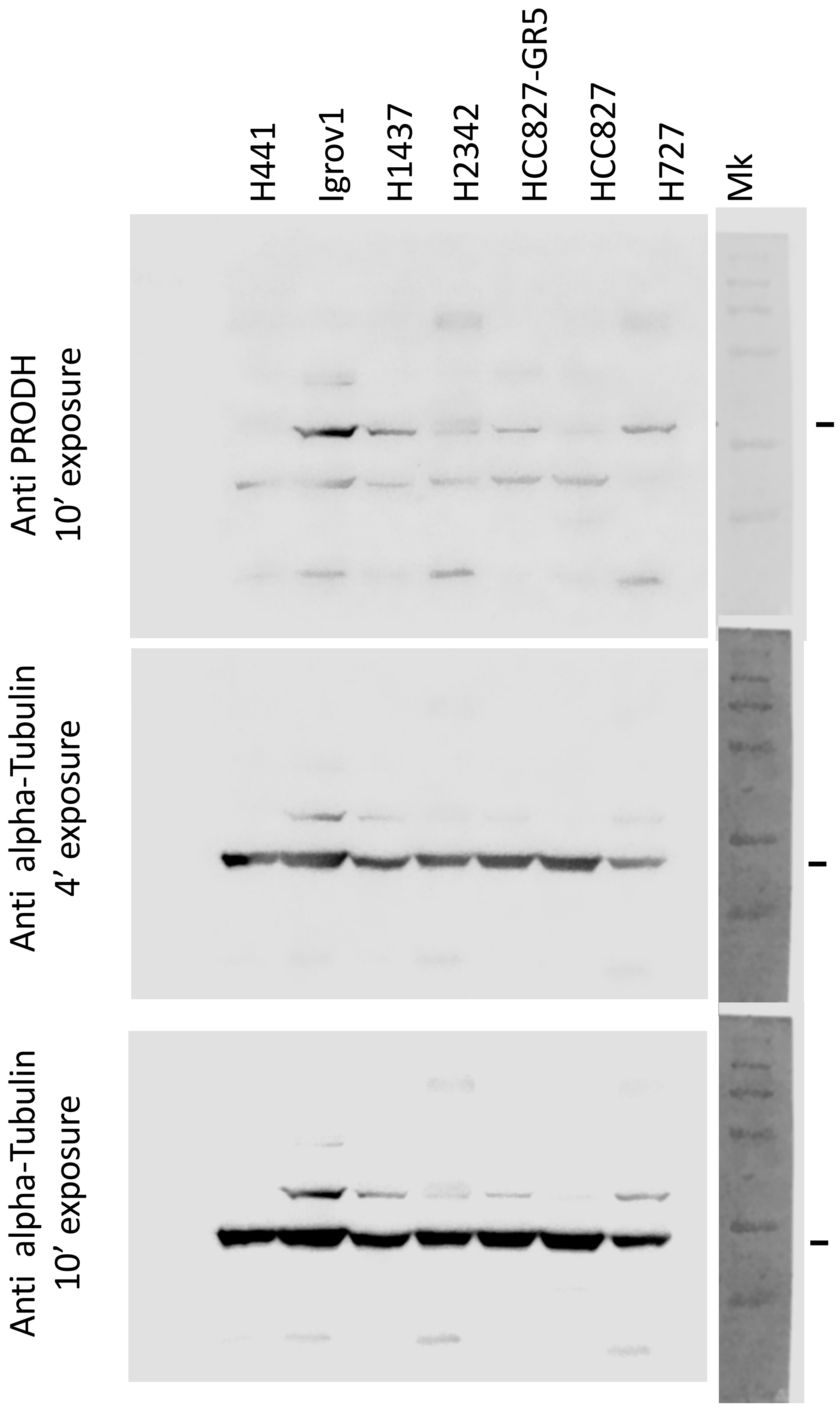

Figure S2.
Raw data of the immunoblots presented in Figure 2, right panel

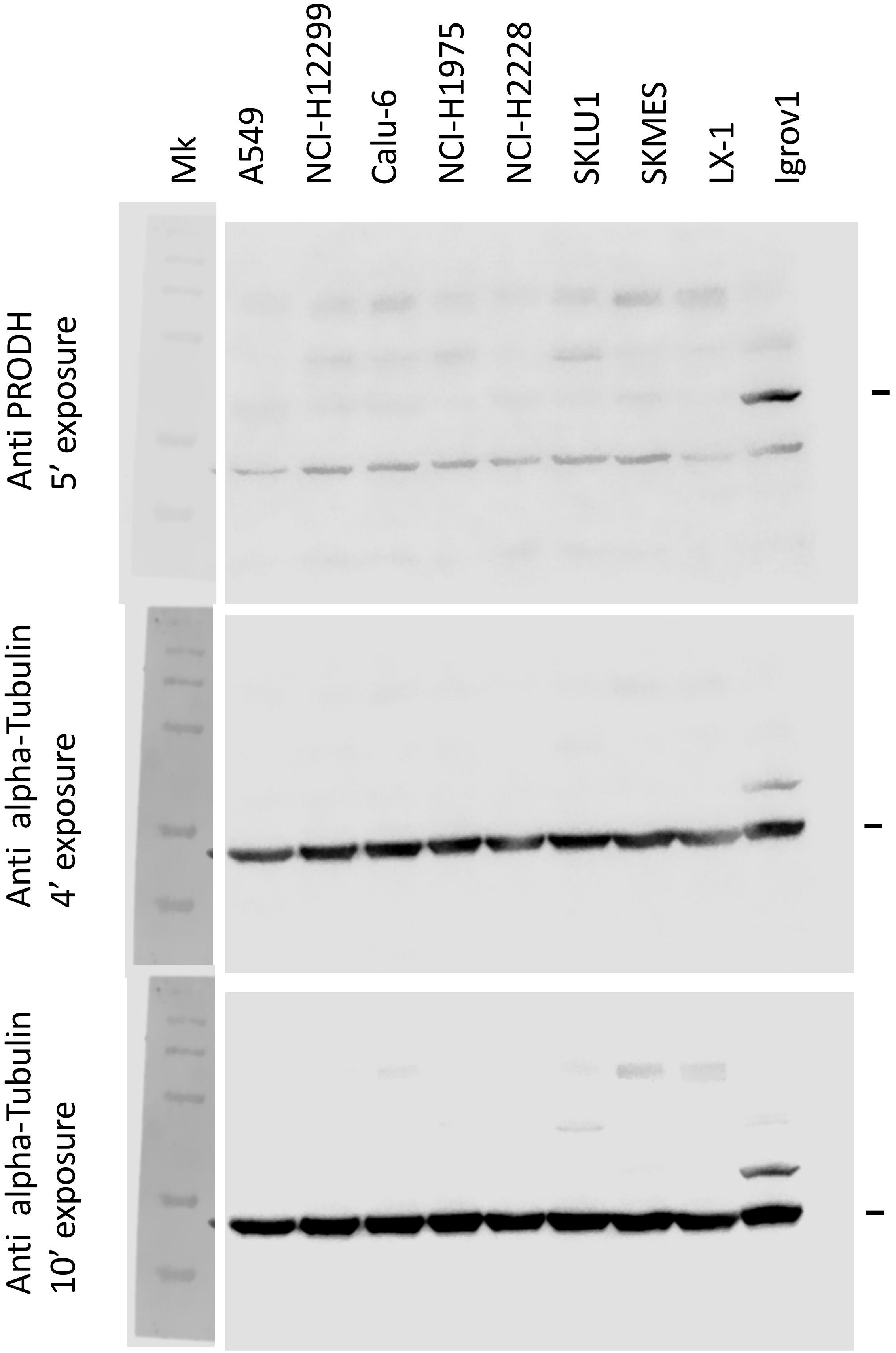

Figure S3.
Raw data of the immunoblots presented in Figure 5

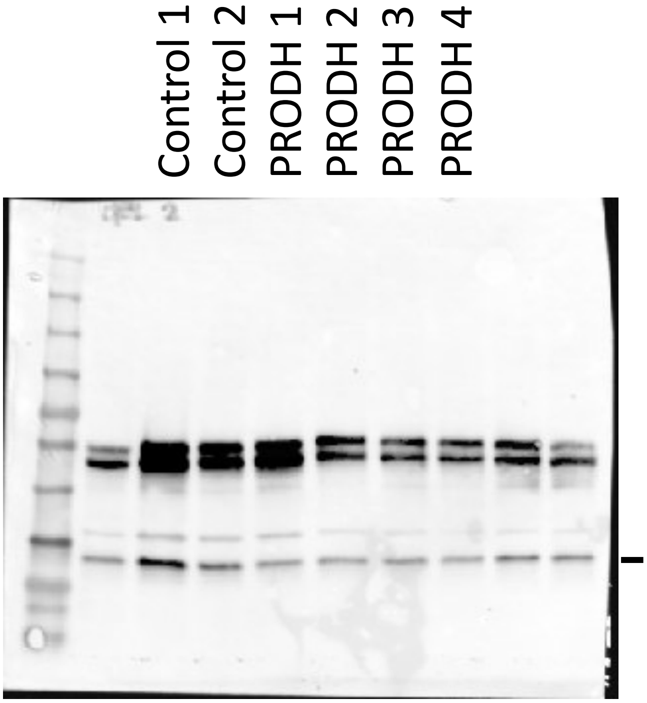

Anti caspase-3

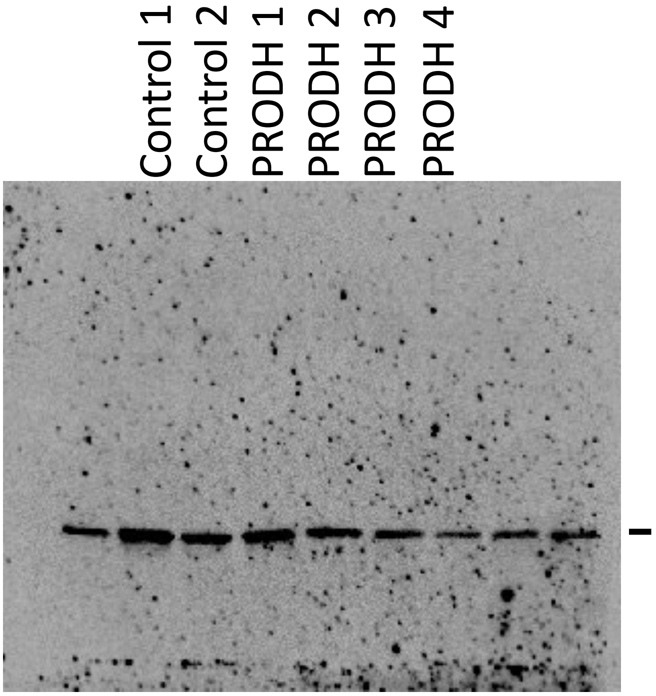

Anti GAPDH
