# Supplementary material for: Proline Dehydrogenase (PRODH) Is Expressed in Lung Adenocarcinoma and Modulates Cell Survival and 3D Growth by Inducing Cellular Senescence
Source: Int J Mol Sci. 2024 Jan 5;25(2):714. doi: 10.3390/ijms25020714 (PMC10815008; doi:10.3390/ijms25020714)
Supplement: Supplementary file 1 [file ijms-25-00714-s001.zip › Supplementary figures S4 heatmap & IL12 secretome.pdf]

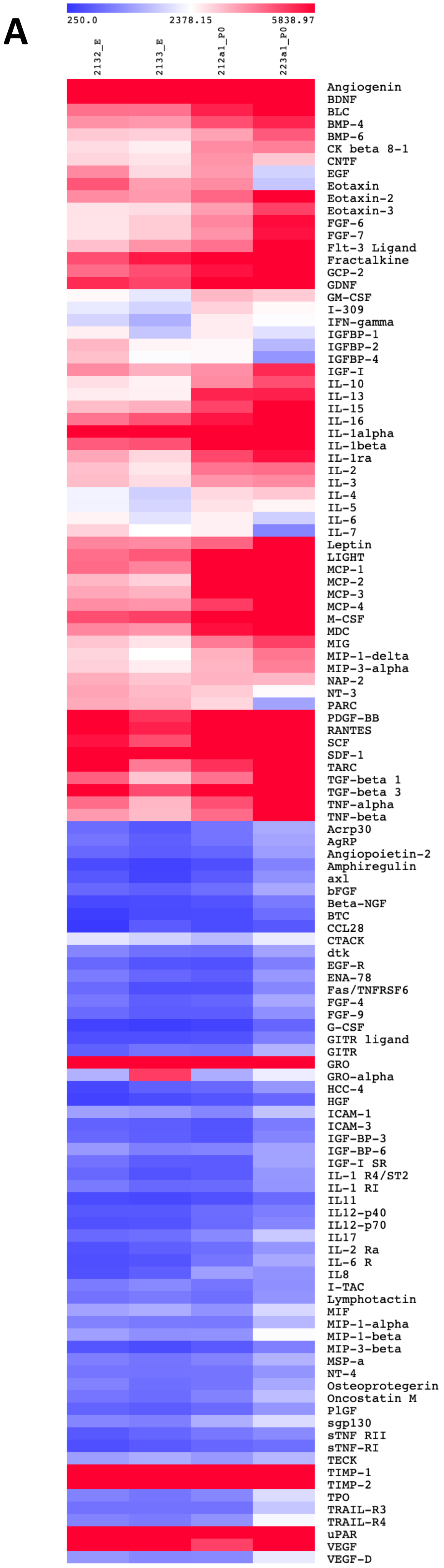

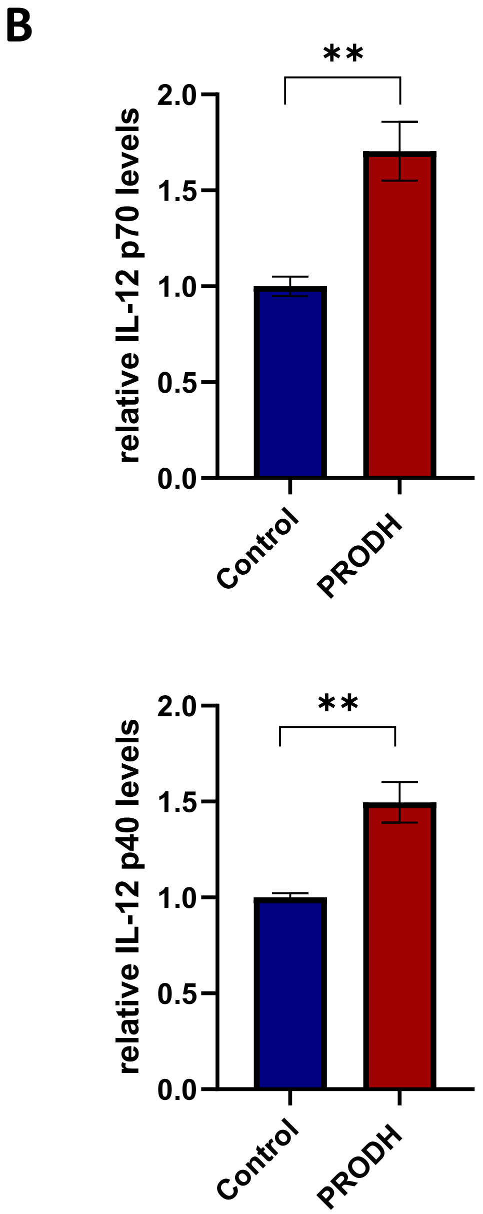

Figure S4. Secretome analysis by antibody array. (A) Heatmap showing the levels of expression of the indicated cytokines as obtained by secretome analysis. From left to right: control clone 1, control clone 2, PRODH clone 1, and PRODH clone 4. **(B) secreted protein levels of the anti-tumor cytokine IL-12 (upper panel IL-12p70, lower panel IL-12p40) in control and PRODH-expressing clones of the NCI-H1299 cell line, as detected by secretome analysis.** The graphs show the average ± SEM of secretome results obtained from two PRODH-expressing clones and two control clones. Asterisks indicate significant differences in the two types of clones (Student t-test; ** indicates *p*-value < 0,01).
