# Supplementary material for: Proline Dehydrogenase (PRODH) Is Expressed in Lung Adenocarcinoma and Modulates Cell Survival and 3D Growth by Inducing Cellular Senescence
Source: Int J Mol Sci. 2024 Jan 5;25(2):714. doi: 10.3390/ijms25020714 (PMC10815008; doi:10.3390/ijms25020714)

Figure S5.
Scheme of antibodies spotted on the two membranes of the antibody array used for secretome analysis

Membrane C6 scheme

POS	POS	NEG	NEG	Blank	Angiogenin	BDNF	BLC	BMP-4	BMP-6	CK beta 8-1	CNTF	EGF	Eotaxin
POS	POS	NEG	NEG	Blank	Angiogenin	BDNF	BLC	BMP-4	BMP-6	CK beta 8-1	CNTF	EGF	Eotaxin
Eotaxin-2	Eotaxin-3	FGF-6	FGF-7	Flt-3 Ligand	Fractalkine	GCP-2	GDNF	GM-CSF	I-309	IFN-gamma	IGFBP-1	IGFBP-2	IGFBP-4
Eotaxin-2	Eotaxin-3	FGF-6	FGF-7	Flt-3 Ligand	Fractalkine	GCP-2	GDNF	GM-CSF	I-309	IFN-gamma	IGFBP-1	IGFBP-2	IGFBP-4
IGF-I	IL-10	IL-13	IL-15	IL-16	IL-1alpha	IL-1beta	IL-1ra	IL-2	IL-3	IL-4	IL-5	IL-6	IL-7
IGF-I	IL-10	IL-13	IL-15	IL-16	IL-1alpha	IL-1beta	IL-1ra	IL-2	IL-3	IL-4	IL-5	IL-6	IL-7
Leptin	LIGHT	MCP-1	MCP-2	MCP-3	MCP-4	M-CSF	MDC	MIG	MIP-1-delta	MIP-3-alpha	NAP-2	NT-3	PARC
Leptin	LIGHT	MCP-1	MCP-2	MCP-3	MCP-4	M-CSF	MDC	MIG	MIP-1-delta	MIP-3-alpha	NAP-2	NT-3	PARC
PDGF-BB	RANTES	SCF	SDF-1	TARC	TGF-beta 1	TGF-beta 3	TNF-alpha	TNF-beta	Blank	Blank	Blank	Blank	POS
PDGF-BB	RANTES	SCF	SDF-1	TARC	TGF-beta 1	TGF-beta 3	TNF-alpha	TNF-beta	Blank	Blank	Blank	Blank	POS

Membrane C7 scheme

POS	POS	NEG	NEG	Blank	Acrp30	AgRP	Angiopoietin-[illegible]	Amphiregulin	axl	bFGF	Beta-NGF	BTC	CCL28
POS	POS	NEG	NEG	Blank	Acrp30	AgRP	Angiopoietin-[illegible]	Amphiregulin	axl	bFGF	Beta-NGF	BTC	CCL28
CTACK	dtk	EGF-R	ENA-78	Fas/TNFRSF6	FGF-4	FGF-9	G-CSF	GITR ligand	GITR	GRO	GRO-alpha	HCC-4	HGF
CTACK	dtk	EGF-R	ENA-78	Fas/TNFRSF6	FGF-4	FGF-9	G-CSF	GITR ligand	GITR	GRO	GRO-alpha	HCC-4	HGF
ICAM-1	ICAM-3	IGF-BP-3	IGF-BP-6	IGF-I SR	IL-1 R4/ST2	IL-1 RI	IL11	IL12-p40	IL12-p70	IL17	IL-2 Ra	IL-6 R	IL8
ICAM-1	ICAM-3	IGF-BP-3	IGF-BP-6	IGF-I SR	IL-1 R4/ST2	IL-1 RI	IL11	IL12-p40	IL12-p70	IL17	IL-2 Ra	IL-6 R	IL8
I-TAC	Lymphotactin	MIF	MIP-1-alpha	MIP-1-beta	MIP-3-beta	MSP-a	NT-4	Osteoproteger	Oncostatin M	PlGF	sgp130	sTNF RII	sTNF-RI
I-TAC	Lymphotactin	MIF	MIP-1-alpha	MIP-1-beta	MIP-3-beta	MSP-a	NT-4	Osteoproteger	Oncostatin M	PlGF	sgp130	sTNF RII	sTNF-RI
TECK	TIMP-1	TIMP-2	TPO	TRAIL-R3	TRAIL-R4	uPAR	VEGF	VEGF-D	Blank	Blank	Blank	Blank	POS
TECK	TIMP-1	TIMP-2	TPO	TRAIL-R3	TRAIL-R4	uPAR	VEGF	VEGF-D	Blank	Blank	Blank	Blank	POS

Figure S6. Membrane pictures used for raw analysis of the secretome

H1299_control 1
(2132_empty)

C6

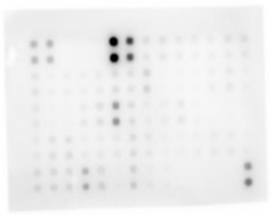

C7

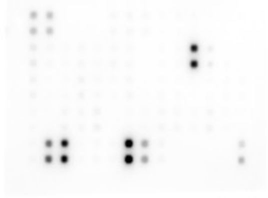

H1299_control 2
(2133_empty)

C6

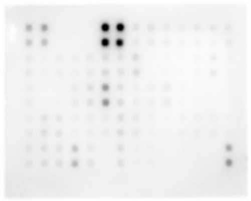

C7

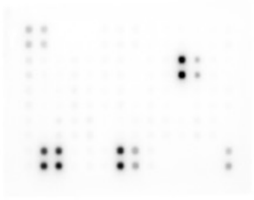

H1299_PRODH 1
(212a1 PO)

C6

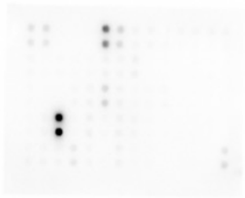

C7

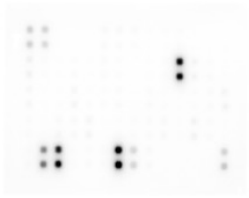

H1299_PRODH 4
(223a1 PO)

C6

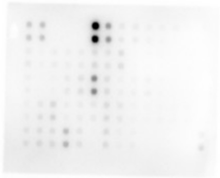

C7

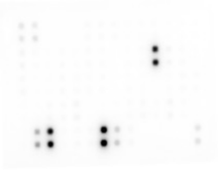

Figure S7. Pictures for data acquisition during secretome analysis

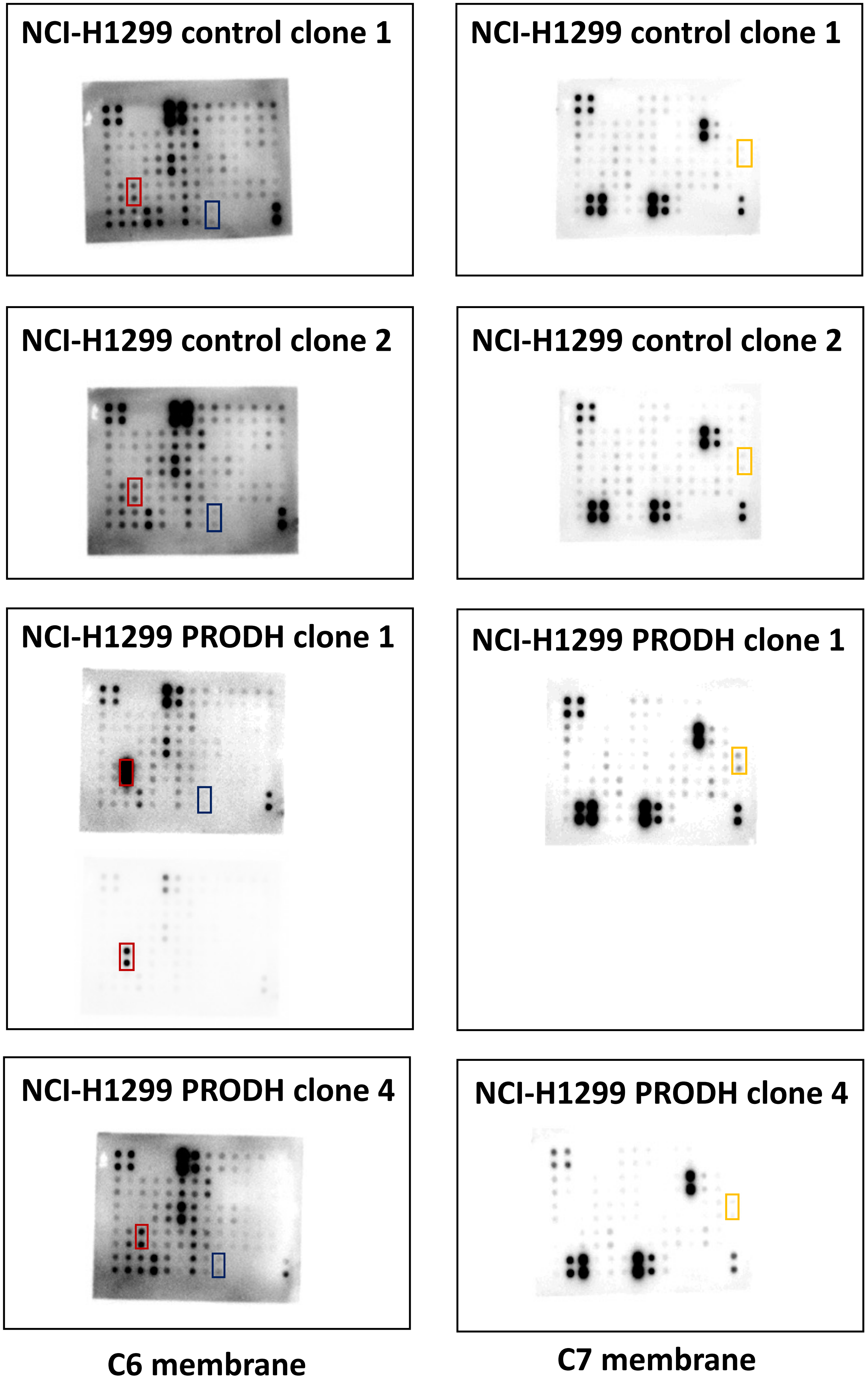

Supplement: Supplementary file 1 [file ijms-25-00714-s001.zip › Supplementary figures S5-S7 secretome raw data & acquisition.pdf]
